# Transfusion transmitted infections among blood donors of Kamenge Teaching Hospital blood bank in Burundi

**DOI:** 10.4314/ahs.v24i1.12

**Published:** 2024-03

**Authors:** Epipode Ntawuyamara, Astere Manirakiza, Ferdinand Nduwimana, Arnaud Iradukunda, Ramadhan Nyandwi, Dionys Nsanzabagenzi

**Affiliations:** 1 Department of Dermatology and Venereology, Faculty of Medicine, Kamenge Teaching Hospital of University of Burundi, P. Box 1020, Bujumbura, Burundi; 2 Department of Dermatology, Cosmetology and Venereology, Shenzhen Hospital of Southern Medical University, Shenzhen, Guangdong, China; 3 Service of Oncology, Department of Internal Medicine, Kamenge Teaching Hospital, Burundi; 4 Doctoral School of University of Burundi, Bujumbura, Burundi; 5 Department of Clinical Biology, Faculty of Health Sciences and Medical Skills, Hope Africa University, Bujumbura, Burundi; 6 Department of Medicine, University of Burundi, Bujumbura, PB 1550, Burundi; 7 Department of Statistics, Lake Tanganyika University, Mutanga, PB 5304, Burundi; 8 Royal Society of Tropical Medicine and Hygiene, 303-306 High Holborn, London, UK; 9 Department of research and Innovation, ARNECH Research and Consulting Office, Burundi; 10 Department of Laboratories Kamenge Teaching Hospital of University of Burundi, Bujumbura, Burundi; 11 Department of Nephrology, Zhujiang Hospital of Southern Medical University, Guangzhou, China; 12 Department of Nephrology, Kamenge Military Hospital, Bujumbura, Burundi

**Keywords:** Transfusion transmitted infections, prevalence, blood donors, blood bank, Burundi

## Abstract

**Background:**

The current risk of contracting a transfusion transmitted infections (TTIs) is unknown in Burundi.

**Objectives:**

The aim of this study was to assess sociodemographic profiles of blood bank donors at Kamenge Teaching Hospital, the prevalence and associated risk factors of HIV, syphilis, HBV and HCV from 2015 to 2020.

**Methods:**

We conducted a cross-sectional study including all blood donors of Kamenge Teaching Hospital blood bank. During this study, 1370 blood samples were screened for HIV, Syphilis, HBV and HCV. We calculated prevalence of TTIs and performed logistic regression to know associated risk factors.

**Results:**

Blood donors were males at 77% and 23% females. They were mostly students (54.2%). On screening, 83 blood samples (6.06%) were seropositive for at least one TTI. The overall prevalence rate of HIV, Syphilis, HBV and HCV among blood donors was 1.3%, 0.2% ,1.6%, 2.9% respectively. There was difference in distribution of the four TTIs among blood donors which is statistically significant (x^2^=33.997, ϱ-value<0.001). Private donors were associated with a high risk of syphilis and being a first-time donor was associated with a high HBV risk factor.

**Conclusion:**

The prevalence of TTIs found still to be high; mandatory and continuous screening is necessary.

## Introduction

In matter of blood transfusion, Human Immunodeficiency Virus (HIV), Hepatitis B virus (HBV) and Hepatitis C virus (HCV) take the name of Transfusion Transmitted Infections (TTIs). TTIs are infections resulting from the introduction of a pathogen into a person through blood transfusion. A wide variety of pathogens, including bacteria, viruses, prions, and parasites can be transmitted through blood transfusions 1. Infectious agents involved are very diverse and include HBV, HCV, HIV-1/2, human T-cell lymphotropic (HTLV-I/II), Cytomegalo- (CMV), Parvo- B19, West Nile (WNV) and Dengue viruses. Trypanosomiasis, malaria, and Transmissible Spongiform Encephalopathy (TSE) also belong to TTIs^2^.

Blood transfusion is the process of transferring blood products into one's circulation intravenously. Although blood transfusion plays an important role in supportive care of medical and surgical patients, unsafe transfusion practices also put millions of people at risk of TTIs^3^. Use of unscreened blood transfusion keep the patient at risk of acquiring many TTIs like HBV, HCV and HIV. This, however can be managed through the elimination of commercial blood donors, a greater monitoring of voluntary donors and a mandatory pre-transfusion evaluation of blood units for HIV, Hepatitis B and C and syphilis., malaria etc.^4^. Preventing the transmission of infectious diseases through blood transfusion in developing countries is difficult given that the resources required are not always available^5^. Blood screening should be performed according to quality system requirements 6.

WHO recommends that all blood donations should be screened for infections prior to use. Screening for HIV, hepatitis B, hepatitis C, and syphilis should be mandatory 6. Burundi is located in a tropical climate where infectious diseases in general and TTIs in particular have a heavy burden on health system.

The aim of this study is to determine the sociodemographic profile of blood donors of Kamenge Teaching Hospital (KTH) blood bank, the prevalence and associated risk factors with HIV, Syphilis, HBV and HCV from 2015 to 2020 among blood donors of KTH Blood Bank in Burundi.

Indeed, the results from this study will constitute a scientific basis, which will help policy makers for improving blood transfusion quality and so healthcare quality in Burundi.

## Methods

### Study design, area and duration

We performed a cross-sectional study using data obtained on 1370 blood units collected during a period of six (6) years from 1 January 2015 to 31 December 2020 at KTH Blood Bank. Among sociodemographic characteristics we studied gender, age, occupation, residence, marital status type of blood donor. In occupation, we included officials who are individuals with professional qualification such health workers, teachers, engineers, employees. Security forces included soldiers and policemen. Private workers were people with low educational qualifications such drivers, technicians, businessmen, etc. Individuals who declared that they had no occupation or those who were engaged in domestic chores at home were captured under occupational category “None”.

### Blood collection and laboratory test

Blood donors were either volunteers, relatives or friends of recipients. Prior to blood sampling, a health professional took history on donors' health status to exclude all infectious diseases. Individuals were required to answer panel of questions on sociodemographic data, previous illness, chronic disease, history of blood transfusion. Questions were targeted to ascertain risky sexual behaviours and to verify individual's marital status, use of barrier contraceptive were asked. In addition, height, weight, blood pressure, and body temperature were measured. Donors with a history of hepatitis or jaundice, under 16 years old, under 50 kg, with hypo /hypertension, women during menstruation, recent unprotected intercourse were deferred. After a physical examination, donors who fulfilled inclusion criteria were allowed to donate 350 mL blood. Samples were collected from every blood unit and sent to the National Centre of Blood Transfusion (CNTS) laboratory for screening. Five (5) ml of blood were drawn from each blood unit; sera were separated, acquired, labelled within two hours of collection and stored at a temperature between 2 and 8°C. They were examined using ARCHITECT i2000SR immunoassay analyser. All the standard protocols were followed. The TTIs for which blood was screened were hepatitis B surface antigen (HBsAg), HCV, HIV-1 and -2 and syphilis using following reagents: Genscreen ULTRA HIV Ag-Ab (Bio-Rad, France), screening kit for the detection of HIV P24 antigen and antibodies to HIV -1 and HIV-2. Monolisa HBsAg ULTRA (Bio-Rad, France) for the detection of the surface antigen of the Hepatitis B virus (HBs Ag).

INNOTEST HCV Ab IV (Fuji Rebio Europe NV, Technologiepark Belgium) for the qualitative detection of antibodies to HCV.

Rapid Plasma Reaginin-Carbon (RPR-Carbon, Cypress Diagnostics, Langdorp, Belgium) for the detection of syphilis.

### Data source and analysis

We collected data from KTH blood bank records covering the period between 1^st^ January 2015 and 31^st^ December 2020. Collected data were checked for completeness, cleaned, coded, and entered into Microsoft Excel spreadsheet before exported into IBM SPSS statistics software version 25 (SPSS, Inc., Chicago, IL) for analysis. Data analysis was three phased: univariate for descriptive analysis followed by bivariate analysis to test significance using chi-square test then finally multivariable analysis where we put in the model only the significant variables identified with significance in bivariate analysis. The ϱ-values were calculated employing the chi-squared test package. We considered a ϱ-value less than 0.05 for a statistically significant test. Furthermore, we performed a multivariable logistic regression to identify potential risk factors associated to TTIs.

### Ethical approval

We obtained authorization to carry out this study from the high authority of KTH. The Human Research Ethics Committee of the Faculty of Medicine, University of Burundi has approved the study protocol. Blood donors implicitly consented before blood donation. Indeed, most of them were voluntary donors. Moreover, data collected did not contain any personal distinguishable information. The study was conducted in accordance with the guidelines of the Declaration of Helsinki.

## Results

### Sociodemographic characteristics of blood donors

We analysed data from 1370 blood samples of blood units collected from1^st^ January 2015 and 31^st^ December 2020 at KTH Blood Bank. Of these ,77% were from male donors. Considering age group, majority of donors (67.4%) were from age group of 25-34 years, and the mean age of donors was 32 .5 years (range 19-67 years) (Tables 2 and 3). A great number of donors were students (54.2%), security forces were less represented (3.4%). Of the donors, repeat time donors represented 75.6% and 24.4% first time donors. Blood donors were single as marital status at 73.6%, from urban area at 97.4% whereas 2.6% are from rural area.

After screening, 83 (6.06%) were seropositive for at least one transfusion transmitted infection. Forty samples (2.90%) were found seropositive for HCV, 22 (1.6%) for HBV, 18 (1.3%) for HIV and 3 samples (0.20%) were positive for syphilis. There was difference in distribution of the four TTIs among blood donors which is statistically significant (x^2^=33.997, ϱ-value<0.001) (Table 1). The most prevalent among the four TTIs is HCV, the less prevalent being syphilis.

**Table 1:** Prevalence of TTIs among blood donors

TTI	Positive	%	Negative	%	χ^2^	P value
HIV	18	1.3	1352	98.7	33.997	<0.001
Syphilis	3	0.2	1367	99.8		
HBV	22	1.6	1348	98.4		
HCV	40	2.9	1330	97.1		
**Total**	**83**	**6.06**	**1287**	**93.94**		

### HIV

The difference in the distribution of HIV in different age groups (x^2^=9.584, ϱ-value=0.020) was statistically significant. A highest proportion was found among blood donors under 24 years (4.2%) whereas the lowest (0.8%) was found in the donors between 25 and 34 years old. At the same time, the difference in the results of HIV according to year of blood donation was statistically significant (x^2^=13.51, ϱ-value=0.019). A high proportion was observed in 2019 (3.10%), followed by 2020 (2.10%) and 2017 (1.8%), to end with the year of 2015 when no HIV positive case was found. Moreover, no difference observed in prevalence of HIV among blood donors according to their gender (x^2^=0.0006, ϱ-value=0.938), occupation (x^2^=7.277, ϱ-value=0.122) or residence (x^2^=0.083, ϱ-value=0.149) (Table 2).

**Table 2:** HIV and Syphilis among blood donors in different sociodemographic characteristics

Sociodemographic characteristics		Total n=13 70	HIV				Syphilis				

			Positive		Negative		Positive		Negative		
			18		1352		3 (0.2%)		1367 (99.8%)		
			(1.3%)		(98.7%)						

		N	%	N	(%)	N	(%)	N	(%)	N	(%)
**Gender**	Male	1055	77	14	1.3	1041	98.7	2	0.2	1053	99.8
	Female	315	23	4	1.3	311	98.7	1	0.3	314	99.7
			?^2^ = 0.006, ϱ=0.938				?^2^ = 0.182; ϱ =0.670				
**Age group**	= 24	95	6.9	4	4.2	91	95.8	0	0.0	95	100
	25-34	923	67.4	7	0.8	916	99.2	2	0.2	921	99.8
	35-44	254	18.5	5	2.0	249	98.0	1	0.4	253	99.6
	45-54	50	3.6	1	2.0	49	98.0	0	0.0	50	100
	=55	48	3.5	1	2.1	47	97.9	0	0.0	48	100.0
			?^2^ = 9.584; ϱ =0.048*				?^2^ = 0.779; ϱ =0.941				
**Occupation**	Official	211	15.5	6	2.8	205	97.2	1	0.5	210	99.5
	Security	46	3.4	1	2.2	45	97.8	0	0.0	46	100.0
	Student	743	54.2	10	0.1	733	99.9	1	0.1	742	99.9
	Private	114	8.3	0	0	255	100.0	1	0.9	113	99.1
	None	256	18.7	1	0.4	114	99.6	0	0.0	256	100.0
			?^2^ = 7.277; ϱ =0.122				?^2^ = 3.793; ϱ =0.043*				
**Marital status**	Married	360	26.3	6	1.7	354	98.3	0	0.0	360	100.0
	Single	1008	73.6	12	1.2	996	98.8	3	0.3	1005	99.7
	Divorced	2	0.1	0	0	2	100.0	0	0.0	2	100.0
			?^2^ = 0.491; ϱ =0.782				?^2^ = 1.080; ϱ =0.583				
**Residence**	Urban	1334	97.4	18	1.3	1316	98.7	3	0.2	1331	99.8
	Rural	36	2.6	0	0	36	100.0	0	0.0	36	100.0
			?^2^ = 0.492; ϱ =0.483				?^2^ = 0.081; ϱ =0.776				
**Type of donor**	First time	334	24.4	7	2.1	327	97.9	2	0.6	332	99.4
	Repeat time	1036	75.6	11	1.1	1025	98.9	1	0.1	1035	99.9
			?^2^ = 2.083; ϱ=0.149				?^2^ = 2.916; ϱ =0.088				
**Year of blood donation**	2015	223	16.3	0	0	223	100.0	0	0.0	223	100.0
	2016	225	16.4	1	0.4	224	99.6	1	0.4	224	99.6
	2017	222	16.2	4	1.8	218	98.2	0	0.0	222	100.0
	2018	248	18.1	1	0.4	247	99.6	1	0.4	247	99.6
	2019	257	18.8	8	3.1	249	96.9	1	0.4	256	99.6
	2020	195	14.2	4	2.1	191	97.9	0	0.0	195	100.0
			?^2^ = 13.51; ϱ =0.019*				?^2^ = 2.654; ϱ=0.753				

*: statistically significant; n: number; %: percentage, **?2:** Chi-square; ϱ: ϱ-value

### Syphilis

There was difference in distribution of syphilis among blood donors according to their occupation which was statistically significant (x^2^=3.793, ϱ-value=0.043). The highest proportion was observed among private workers (0.90%) followed by first time blood donors (0.60%) (Table 2).

### HBV

There was a difference in distribution of HBV (x^2^=5.387, ϱ-value=0.020) among first time (3%) and repeat time donors (1.20%) which is statistically significant. A high prevalence was observed in security workers (4.30%) without showing a statistically significant difference compared to other categories of occupation (x^2^ =4.725; ϱ=0.317) (Table 3).

**Table 3:** HBV and HCV among blood donors in different sociodemographic characteristics

Sociodemographic characteristics		Total n=1370		HBV				HCV			

				Positive		Negative		Positive		Negative	
				22		1348		40 (2.9%)		1330 (97.1%)	
				(1.6%)		(98.4%)					
		**N**	**%**	**N**	**%**	**N**	**(%)**	**N**	**%**	**N**	**%**
**Gender**	Male	1055	77	20	1.9	1035	98.1	28	2.7	1027	97.3
	Female	315	23	2	0.6	313	99.4	12	3.8	303	96.2
				?2 =2.44; ϱ=0.118				?2 =1.143; ϱ=0.285			
**Age group**	= 24	95	6.9	2	2.1	93	97.9	5	5.3	90	94.7
	25-34	923	67.4	14	1.5	909	98.5	26	2.8	897	97.2
	35-44	254	18.5	5	2.0	249	98.0	6	2.4	248	97.6
	45-54	50	3.6	1	2.0	49	98.0	2	4.0	48	96.0
	=55	48	3.5	0	0	48	100.0	1	2.1	47	97.9
				?2 =1.240; ϱ=0.871				?2 =2.478; ϱ=0.649			
**Occupation**	Official	211	15.5	2	0.9	209	99.1	11	5.2	200	94.8
	Security	46	3.4	2	4.3	44	95.7	0	0	46	100.0
	Student	743	54.2	13	1.7	730	98.3	23	3.1	720	96.9
	Private	114	8.3	3	2.6	111	87.4	1	0.9	113	99.1
	None	256	18.7	2	0.8	254	99.2	5	2.0	251	98.0
				?2 =4.725; ϱ=0.317				?2 =7.902; ϱ=0.095			
**Marital status**	Married	360	26.3	5	1.4	355	98.6	7	1.9	353	98.1
	Single	1008	73.6	17	1.7	991	98.3	33	3.3	975	96.7
	Divorced	2	0.1	0	0.0	2	100.0	0	0.0	2	100.0
				?2 =0.181; ϱ=0.913				?2 =1.714; ϱ=0.424			
**Residence**	Urban	1334	97.4	21	1.6	1313	98.4	40	3.0	1294	97.0
	Rural	36	2.6	1	2.8	35	97.2	0	0	36	100
				?2 =0.321; ϱ=0.571				?2 =1.112; ϱ=0.292			
**Type of donor**	First time	334	24.4	10	3.0	324	97.0	14	4.2	320	95.8
	Repeat time	1036	75.6	12	1.2	1024	98.8	26	2.5	1010	97.5
				?2 =5.387; ϱ=0.020*				?2 =2.521; ϱ=0.112			
										
Year of blood donation	2015	223	16.3	6	2.7	217	97.3	0	0.0	223	100.0
	2016	225	16.4	2	0.9	223	99.1	5	2.2	220	97.8
	2017	222	16.2	4	1.8	218	98.2	10	4.5	212	95.5
	2018	248	18.1	2	0.8	246	99.2	8	3.2	240	96.8
	2019	257	18.8	5	1.9	252	98.1	8	3.1	249	96.9
	2020	195	14.2	3	1.5	192	98.5	9	4.6	186	95.4
				?2 =3.643; ϱ=0.602				?2 =11.154; ϱ=0.048*			

*: statistically significant; n: number; %: percentage, **?2: Chi-square;** ϱ: ϱ-value.

### HCV

The difference in distribution which is statistically significant is observed in different years of blood donation (x^2^=11.15, ϱ-value=0.048). The highest prevalence was found in 2020 (4.60%), followed by 2017 (4.50%) and 2018 (3.20%), to finish by 2015 when no case of HBV was found among blood donors (Table 3).

### Prevalence and associated of factors of HIV, Syphilis, HBV and HCV

Every factor which was statistically significant in univariate analysis was subjected to a binary logistic regression model using a standard method. The results of logistic regression analysis revealed a statistically significant association between year of blood donation and HIV (AOR=0.631, ϱ-Value=0.006) and HCV (AOR=0.771, ϱ-Value=0.013). To donate blood in 2017 was associated with a higher risk of HIV (1.80%) and HCV (4.50%). Significant differences exist also between occupation and syphilis (AOR=25.391, ϱ-Value=0.037), and between type of blood donor and HBV (AOR=0.347, ϱ-Value=0.017). Private donors were associated with a high risk of syphilis and being a first-time donor was associated with a high HBV risk factor (Table 4).

**Table 4:** Final model of factors associated with positive HIV, Syphilis, HBV and HCV among blood donors

TTI	Characteristic	AOR	95% CI	ϱ –Value
**HIV**	Year	0.631	0.451-0.878	0.006
**Syphilis**	Official	3.165	0.158-63.486	0.451
	Private	25.391	1.224-526.592	0.037
	None	Ref		
**HBV**	First time	0.347	0.145-0.828	0.017
	Repeat time	Ref		
**HCV**	Year	0.771	0.629-0.946	0.013

## Discussion

### Sociodemographic profile of blood donors

In our study, the male predominance in blood donation may be explained by a general belief that men are healthier than women, and thus are more suitable for blood donation 7,8. It may be also explained in part by some physiological status of women like menstruation, pregnancy and breast feeding, which temporarily prohibited the females from the blood donation 9. Moreover, women have lower hemoglobin levels and a higher number of vasovagal reactions. This may cause a high rate of refusal as women donors 8. This participation of women in blood donation at KTH Blood Bank is similar to that found by M.B. Nagalo et al.24.38%, Abebe et al.29.9% and the proportion of female blood donors (33%) according to WHO 7,8,10. It is highest compared to that of Talal Alharazi et al., Mohammed et al., D. M. Doungous et al. who found 0.9%,2% and 3.9%, successively 11–13. The minimum and maximum age were 19 and 67 years with the mean age of 32.5years. The group age of 25-34 years constitutes the first represented age group at 67.4% followed by 35-44 years (18.5%). This result is similar to the study conducted in Yemen where the vast majority of blood donors were aged between 26–35 years (48.70%) 14. This proportion of blood donors is high compared to the publication of WHO which states that 40% of donations were given by donors aged 25–44 years globally and 42% of blood donations were given by donors aged below 24 years in low- income countries 15. This is due to the fact that the population of Burundi in general, Bujumbura city in particular is very young as blood donors are from the general population 16.

In this study, most of blood donors were voluntary, unpaid donors. We did not find any paid donors. The same results have been found by Legese et al.in Ethiopia 14. Regular, voluntary, unpaid blood donors can assure an adequate and reliable supply of safe blood. These are the safest groups of donors as the prevalence of blood borne infections is lowest among them 7,8.

Many of blood donors at KTH Blood Bank were students. Those are secondary school students and university students who have good willing to save lives by blood donation. Indeed, KTH being a teaching hospital, some of blood donors are students or trainees who come to study medicine and allied sciences at that hospital. The most common reason that impels students to donate blood is the desire to help another human being 17. Students ‘predominance also has been found by Alsughayyir in Saudia Arabia at 49.40% 18.

Many of blood donors (75.60%) where repeat time donors while 24.40% were first time donors. These results are similar to these found in 2021 at CNTS 19 where repeat time donors represented 73.45%- and first-time donors at 26.51%.

### Prevalence of transfusion transmitted infections

This study showed that 6.06% of blood donors were seropositive for at least one of the screened transfusion-transmitted infections. Considering the overall prevalence, there is difference in distribution of TTIs which is statistically significant (x^2^=33.997, ϱ-value<0.001). The prevalence was higher for HCV (2.90%) followed by HBV (1.60%), HIV (1.30%) for closing with Syphilis (0.2%). Our finding was lower when compared to study done by Yacob et al. (20), Alex et al. 21, Babiker et al. 22who found 12.9 %,10.1 % and 22.52 %, successively. The reason for the lower rate of prevalence of TTI in this study compared with other studies may be due to the efficacy of the screening technique used before blood taking and also may be because of existence of different magnitude of risk factor for contracting transfusion transmitted infection. In the study done in the same country at national transfusion centre, the prevalence was almost the same where 5.94% had at least one TTI 19. The prevalence of TTIs was higher than the findings from Elias et al. 23, TTI in Eritrea by Nejat et al. 24, Ethiopia by Legese et al.^25^ which are 6.00%,3.60 % and 5.40% successively. The higher prevalence in our case may be due to the difference in the health care system in different study setting. The difference in prevalence of HIV among donors in relation to the age group was found to be statistically significant (x^2^=9.584, ϱ-value=0.020). This prevalence is similar to HIV prevalence in the population above 15 years in Burundi according to 2020 HIV spectrum modelling in Burundi 26. This prevalence is higher than those found by Bhatti et al. 27 in Pakistan in 2022(0.01%) and Ling Shi et al. 28 in China in 2020 (0.06%). This is because even the health system in these countries is stronger than in Burundi.

The HBV prevalence found in our study is close to that found by Kwizera et al. 1.04% in Burundi 29 in 2018 and Arshad et al. 1.84% in Pakistan 30. Ntawuyamara and Daxing 19 at CNTS in Burundi found HBV's prevalence at 1.68%. R. Ntagirabiri et al. in their study estimated the prevalence of HBsAg in general population at 4.60% in Burundi 31. This prevalence is lowest compared to that found by Eric Osei et al. 32, Walana et al. 33, Amiwero et al. 34 which were 7.50%,9.60% and 14.40% successively. Even if blood donors do not constitute a sufficient representative population for estimation of the prevalence of HBV in general population, this study shows that there is good progress in fighting against HBV. Even though, this prevalence is high compared to the countries where hepatitis B vaccination rate is high 35,36.

HCV prevalence was very high (4.20%) in under 24 years old age group and lower (2.10 %) in 55 years old and above This prevalence is different to that found by Kwizera et al. 37 in 2018 who found that HCV prevalence among blood donors at CNTS was 1.12%. CNTS and KTH Blood Banks all together being located in Bujumbura city; this difference can make us think about the increasing of HCV prevalence in Bujumbura general population or Bujumbura blood donors. The prevalence found is relatively higher compared to that found by Abid and Athraa 35 at Samarra's General Hospital (2.40%), Ping et al. 36 among blood donors in China (0.16%) and. Bhatti et al. 27 in Pakistan (1.50%). Indeed, HCV in Burundi is one of the highest rates in Africa. Anti-HCV antibodies was estimated to 8.20% in 2018 in Burundi 38.

None study has been done to evaluate the prevalence of syphilis among general population in Burundi. The prevalence we found is lower than 0.80% by Bhatti et al 27 in Pakistan ,0.88% in Chengdu 39, China;4.70%,3.70% and 1.40% in neighbouring countries of Tanzania and Democratic Republic of Congo 40,41. It is also lower than the prevalence of syphilis of 1.6 % in general population in Africa WHO region 42. The very low prevalence of Syphilis among blood donors who come mainly in Bujumbura city may explain the efficacy in STI prevention and management protocol in Burundi 43.

### Logistic regression analysis of factors associated to transfusion transmitted infections

In our study, HIV and HCV prevalence were associated with the year of blood donation. Age group, gender or residence were determined to be not statistically associated with HIV and HCV. A study conducted in China not using a condom, having sex with HIV-infected individuals, were all associated with more than five times higher odds of having HIV 44. In a study conducted in Mali, gender, age and education level was associated with HCV-infection 45. HBV infection among blood donors was associated with type of blood donors. To be a first time was associated with high risk of HBV. This may be explained by the fact that some first-time donors do not know their serology and prefer to donate blood as a free opportunity to have one's serology tested. This risk factor has been also found by Aude Jary in Mali 45. In our study, syphilis infection was associated by blood donor's occupation. To be a private worker was statistically associated with a high risk of presenting syphilis. Contrary to other studies, syphilis was not associated with group age or occupation 44. In a study done in Jinan China by Chen et al. 46, female, 35-44 years old, with a lower education degree, farmers and first-time donors were syphilis high-risk factors.

## Conclusion

We found a high prevalence of TTIs; continuous blood screening is required. Our study showed the high prevalence of HCV among blood donors. The government should increase effort in HBV vaccination as one strategy to decrease TTIs among blood donors.
